# Black Nativity and Health Disparities: A Research Paradigm for Understanding the Social Determinants of Health

**DOI:** 10.3390/ijerph19159166

**Published:** 2022-07-27

**Authors:** Mosi Adesina Ifatunji, Yanica Faustin, Wendy Lee, Deshira Wallace

**Affiliations:** 1 Departments of African American Studies and Sociology, College of Letters and Science, University of Wisconsin at Madison, Madison, WI 53706, USA; 2 Department of Public Health Studies, College of Arts and Sciences, Elon University, Elon, NC 27244, USA; yfaustin@elon.edu; 3 Department of Sociology, College of Letters and Science, University of Wisconsin at Madison, Madison, WI 54706, USA; wklee3@wisc.edu; 4 Department of Health Behavior, Gillings School of Global Public Health, University of North Carolina at Chapel Hill, Chapel Hill, NC 27599, USA; ddwallac@email.unc.edu

**Keywords:** Blacks, African Americans, Black immigrants, immigrants, health disparities

## Abstract

After more than a century of research and debate, the scientific community has yet to reach agreement on the principal causes of racialized disparities in population health. This debate currently centers on the degree to which “race residuals” are a result of unobserved differences in the social context or unobserved differences in population characteristics. The comparative study of native and foreign-born Black populations represents a quasi-experimental design where race is “held constant”. Such studies present a unique opportunity to improve our understanding of the social determinants of population health disparities. Since native and foreign-born Black populations occupy different sociocultural locations, and since populations with greater African ancestry have greater genetic diversity, comparative studies of these populations will advance our understanding of the complex relationship between sociocultural context, population characteristics and health outcomes. Therefore, we offer a conceptual framing for the comparative study of native and foreign-born Blacks along with a review of 208 studies that compare the mental and physical health of these populations. Although there is some complexity, especially with respect to mental health, the overall pattern is that foreign-born Blacks have better health outcomes than native-born Blacks. After reviewing these studies, we conclude with suggestions for future studies in this promising area of social and medical research.

## 1. Introduction

In recent decades, researchers in the social and medical sciences have revisited a longstanding interest in racialized disparities in population health [1,2,3,4,5]. As it stands, after more than a century of research and debate, the scientific community has yet to reach agreement on the principal causes of these multigenerational disparities. We contend that one of the central reasons for stasis rests within the most prominent study design for investigating these disparities—comparing different racial groups on various measures of health status. This design risks confounding between racial group membership and real or perceived differences in social context and/or population characteristics—factors often left unobserved [6]. That is, the debate on how to interpret race residuals (we are referring to the fact that dummy variables for race in regression models often remain statistically significant after controlling for known correlates of a disease) turns on whether these residuals reflect unobserved differences in the social context or unobserved differences in population characteristics [7,8,9]. Therefore, our understanding of health disparities is incombered by overarching disagreements as to whether “race” is defined by or rooted in social context, group behaviors or biological endowments.

We join others in suggesting that the presence of a growing and diverse foreign-born population in the United States [10,11] represents an opportunity to improve our understanding of the social determinants of racialized population health disparities [12,13,14,15,16,17] (we use the term “foreign-born Blacks” to refer to those born outside the country that are either self-identified or legally classified as Black in the United States). To this end, it is important to note that disparities in morbidity and mortality between native-born Blacks and foreign-born Blacks are larger than nativity differences within any other racialized population—e.g., between native and foreign-born Latinx or Asian populations [18,19,20,21,22] (we use the term “native-born Blacks” to refer to all Blacks born in the United States. This term includes Blacks with foreign-born parents and African Americans, or the decedents of American slaves). Therefore, in addition to improving our descriptive understanding of increasing heterogeneity in Black population health, the fact that native- and foreign-born Blacks share the same racial status but often have different health profiles also presents us with a quasi-experimental design for studying factors that might also contribute to racialized disparities in population health [12,13,14,15,16,17]. That is, since native and foreign-born Blacks share the same racial status while sometimes having different experiences, cultural values and practices [13,23,24], comparative studies of these populations might improve our understanding of the ways in which various race-related sociocultural factors might contribute to disparities in population health between these and other groups [12,13,14,15,16,17].

While there are a range of possible native/immigrant comparisons, the comparison of native and foreign-born Blacks represents a uniquely useful research paradigm because Black people are the only U.S. population whose racial classification has to do with notions of genetic ancestry [25] and also includes a large immigrant population [10,26]. While “blood quantum” has also been a key determinant of being legally classified as Native American [27], there are no Native American “immigrants” in the United States (we understand that there are many Native Americans that migrate from reservations to other locations in the United States and that this experience may be like the experiences of many foreign-national migrants. While scientists should conduct more research on the ways in which this experience shapes Native American health, we reserve the term “immigrant” to refer to the migration of foreign nationals). Conversely, there are large numbers of Asian and Latinx immigrants, but the Census Bureau legally classifies these populations according to their language and/or national origins, not blood quantum or genetic ancestry. Moreover, the current practice of the U.S. Census conceptualizes Latinx as an ethnic group that is composed of different racial population groups. That is, we continue to debate whether people with proximal ancestry in Latin America constitute their own racial group or an ethnic group that includes different racial categories. Given that this debate remains unsettled, we do not include studies that include “Black” migrants from Latin America [28,29,30].

It is also important to note that populations with greater amounts of African genetic ancestry tend to have greater genetic variation [31]. This means that we can gain a more fine-grained analysis of the potential interplay between the environment, genomics (including, single nucleotide polymorphisms, methylation and/or gene expression) and population health, especially since there is simultaneously great variation in the health of these populations [18,22].

We advance the argument for the comparative study of native and foreign-born Blacks in four ways. First, we review differences between these populations vis-à-vis the most prominently studied determinants of population health (i.e., health behaviors, socioeconomic status, environmental factors and population genetics), arguing that since these populations have different social and cultural locations and practices, comparative studies of these populations might yield important insights into the social determinants of population health. Second, we argue that, since native and foreign-born Blacks experience and interpret race differently in the U.S. [24,32,33,34,35,36], the comparative study of these populations will also advance our understanding of the role of racialized bias and discrimination in population health disparities. Third, we suggest that, since these populations share the same racial status, and since populations with greater amounts of African ancestry have greater genetic diversity [31], the comparative study of these populations will improve our understanding of the complex interplay between sociocultural context, population genetics and health outcomes in a way that avoids debates having to do with the nature of racial classification (e.g., [37,38]). Fourth, we encourage the comparative study of native and foreign-born Black health by offering a summary review of 208 existing studies that compare the health of these populations, including potential data sources for further investigation. This is the first review of its kind and offers the current state of knowledge on patterned health differences between native and foreign-born Blacks. We conclude our review with our thoughts on potential future directions within this research paradigm.

## 2. Descriptive Benefits

Most studies of health disparities between White and Black (read native-born Black or African American) populations in the United States have an implied interest in the relative health of native-born Whites and the decedents of enslaved Africans in the United States. However, over the last five or six decades, the country of origin of the U.S. Black population has diversified. Between 1960 and 2005, a 22-fold increase in the number of foreign-born Black people living in the United States occurred (from 125,000 to 2,815,000; [10]). Since 1990, this rapid increase in the size of the foreign-born Black population has been met with increasing diversity in the regions and countries from which immigrants originate. While foreign-born Blacks from the Caribbean have been migrating to the U.S. since the early 1900s [26], between 2000 and 2005, more foreign-born Blacks entered the U.S. from Africa than the Caribbean [10]. Moreover, there is greater diversity in the reasons for migration, especially from African countries—more enter as refugees, asylees or on diversity visas [10]. Black people also migrate to the U.S. from non-majority Black countries throughout Europe [17]. Increases in the size and diversity of the foreign-born Black population in recent decades complicates our understanding of the U.S. Black population [10,24]. That is, increasing portions of Blacks in the United States do not have ancestry in American slavery and are therefore not part of the traditional perspective on multigenerational Black–White inequality in the U.S.

Although these population trends have resulted in greater diversification in the Black population, most research into population health overlooks this diversity. This practice obscures our ability to analyze and understand the increasing variation in the health status of this rapidly diversifying population. Indeed, disparities between native and foreign-born Blacks are wider than those between other native and foreign-born populations within other racialized groups [18,22]. As our review of the literature shows, there are a range of health differences between native and foreign-born Blacks, but most studies show that foreign-born Blacks have better health profiles than native-born Blacks. This means that, in addition to not having a good understanding of the health status of an increasingly diverse Black population, it is likely that studies that do not account for this heterogeneity are underestimating and potentially misunderstanding the basic nature of population health differences between Blacks and Whites. Parsing the Black population will therefore improve our understanding of the nature of Black population health and Black–White health disparities in the United States.

## 3. Perspectives on Black Ethnicity and Population Health

While most are interested in the health of the decedents of American slavery, there is also a longstanding interest in the comparative study of different Black populations as part of a larger effort to understand and unpack the nature of Black–White racial disparities across a range of life domains. These began with distinctions between Blacks, both “slave” and “free” [23,39], but also included distinctions between native and foreign-born Blacks [23,40]. The more recent of these studies show that these populations have different levels and kinds of education [41,42], hold different kinds of jobs [43], attain different levels of success in the labor market [44,45] and often live in different neighborhoods, experiencing different levels of housing segregation from Whites [46,47]. Therefore, while they might share a similar multivariate distribution of skin color, hair texture and craniofacial bone structure, these populations experience the U.S. racialized social system [48] differently [24,32,33,34,35,49,50,51,52,53,54,55]. Differences in sociocultural context and practice matched with similarities in markers that are widely associated with “racial status” mean that the comparative study of these populations presents a quasi-experimental study design whereby racial status is “held constant” while allowing various factors that are associated with the “social determinants of health” to vary [12,13,14,15,16,17].

Population health researchers have previously described the basic advantages of this study design. For instance, in 1992, Richard Davis argued that, given social and cultural differences between native and foreign-born Blacks, there might be great scientific benefit to model stratification across these populations. He argued that “…it stands to reason that the assimilation patterns of native and foreign-born Blacks may be fairly different; if for no other reason than that they often speak a different language… Foreign-born bilingual or non-English speaking Blacks may find it more convenient to coalesce among themselves… these factors suggest the possibility of a very different pattern of assimilation for Black ethnics” [13].

About a decade later, James Jackson and colleagues suggested that comparisons between native and foreign-born Blacks would help refine our understanding of “… the types and amounts of racial and non-racial factors that affect the differential distribution of mental disorders within race and ethnic groups can be identified, leading to possible explications of how race and ethnic group memberships combine with different types of stressors to affect mental health” [16] and that, “… differential immigration experiences among [foreign-born Blacks] will contribute to understanding the mental health implications of racial/ethnic identity and acculturation strategies” [16]. They then suggest that this “… will result in a better understanding of the nature of racial and ethnic differences in the distribution of serious mental disorders” [16].

Shortly thereafter, Carlotta Arthur and Edward Katkin argued that comparative studies of native and foreign-born Blacks will “… raise awareness of the issue of ethnicity among Black populations and to bring attention to new ways to think about health disparities research and the promise that such explorations hold for finding new avenues through which to intervene” [12], including “…in-depth knowledge of the ways in which ethnicity and culture interact with health-related issues in Black populations in the U.S” [13].

While these scholars have done well to describe the ways in which the comparative study of native and foreign-born Blacks will allow for insights into the role of “race and culture” in population health, we develop this perspective further, offering a more detailed consideration of how such studies might improve our understanding of population health disparities. That is, the previously proposed distinctions focus on “race”, “ethnicity” and “culture”, broadly speaking. We offer a more detailed assessment, parsing these broad distinctions into four principal causes of population health: health behaviors, socioeconomic status, environmental stressors and population genetics [15,56]. Since we believe that earlier efforts were an attempt to say that the comparative study of native and foreign-born Black health might allow for a greater understanding of the relative role of these principal causes, we illustrate the utility of this comparative approach by reviewing differences between these populations across the principal causes of health disparities.

### 3.1. Health Behaviors

Most health scientists agree that diet, nutrition and exercise matter for individual health outcomes, but there are still questions concerning the relative importance of health behaviors in explaining racialized differences in population health. Some researchers have speculated that “unexplained differences reflect unmeasured factors that are associated with both race/ethnicity and the specified outcome but are not related to either discrimination or socioeconomic position, [including] culturally shaped patterns of food consumption” [57]. Indeed, studies show that foreign-born Blacks report what some may classify as better health behaviors than native-born Blacks, suggesting that this unobserved “cultural” factor might contribute to health differences between these populations. For instance, native-born Blacks are more likely to smoke than foreign-born Blacks [19,58,59]. Native-born Blacks are also more likely to drink and use drugs than foreign-born Blacks [59,60]. Foreign-born Blacks are more likely than native-born Blacks to report “at least some physical activity” [59], and they have more healthful diets than native-born Blacks [61]. Since these populations have known differences in health behaviors, their health differences might stem from different health behaviors.

### 3.2. Socioeconomic Status

One of the central arguments in studies that focus on the social determinants of health is that socioeconomic status plays a central, if not fundamental, role in shaping population health [62]. Native and foreign-born Blacks also have known differences in socioeconomic status and resources. Foreign-born Blacks have greater educational attainment than native-born Blacks [44] and are also more likely to attend more selective colleges and universities [42]. Although newly arriving foreign-born Blacks often have lower annual earnings than native-born Blacks, their earnings often match or exceed the earnings of native-born Blacks after being in the labor market for about 15 years [44,63,64]. Foreign-born Blacks also have a greater occupational status than native-born Blacks [43]. Since socioeconomic status and resources play a key role in population health disparities and foreign-born Blacks have greater socioeconomic status profiles than native-born Blacks, health differences between these populations might result from differences in socioeconomic status.

### 3.3. Environment

The environmental factors that contribute to health outcomes range from psychosocial stressors to the physical ecology of the built environment. Studies that assess housing segregation between native and foreign-born Blacks show that, while not as pronounced as Black–White segregation, these populations often live in different areas [47,65]. Therefore, there may be some differences in their physical ecology. Although these populations experience similar levels of racial discrimination [34], the nature of these racialized experiences may be different [32,53], and/or they may process or cope with relatively similar experiences differently [24,66,67]. Unlike native-born Blacks, foreign-born Blacks experience various forms of stress associated with the process of immigrant assimilation [32,55], and they enter the United States from both majority-White and majority-Black sending countries [17], which means Black populations in the United States might have historical differences in racial context at different stages of the life course [14,68,69]. Since these populations often live in different neighborhoods and experience different psychosocial stressors (differently), comparative studies might allow for improvements in our understanding of how the social and physical environment contributes to disparities in population health.

### 3.4. Genetics

In recent years, medical scientists have made tremendous headway in furthering our understanding of the links between genetics and individual health outcomes, but the relationship between genetics and health disparities between population groups remains less clear [70,71,72]. Still, some interpret race residuals as offering evidence of the role of unobserved differences in population genetics, which, when comparing Whites and Blacks, may, knowingly or unknowingly, lend credence to arguments for the genetic determinants of racial difference [73] (these scientists are often much less clear or forthcoming about what these genetic differences might be and how they might contribute to differences in health between populations with different racial statuses). Notwithstanding the fact that native and foreign-born Blacks share the same racial status and similar distributions of African genetic ancestry while occupying different sociocultural contexts, comparative studies avoid contentious debates on the genetic basis of racial difference and the potential role of such genetic differences in population health disparities. However, scientists can still further investigate the potential role of genetics in population health (e.g., single-nucleotide polymorphisms, genetic methylation and gene expression; [37,38,74]). For instance, while native and foreign-born Blacks have similar physical features that are widely associated with Blackness (i.e., skin color, hair texture and craniofacial bone structure; [75]), it is entirely possible that these populations have other important biological differences. Indeed, there is more genetic variation in populations with greater African ancestry, which might further advance our understandings of complex interactions between genes, the environment and health. For example, such studies might consider differences in DNA methylation and/or gene expression [76] and how such differences might be related to differences in historical traumas [77,78].

## 4. Methods

Since we believed that, compared to the study of other native and immigrant populations, there were few studies of native and foreign-born Black populations, we began with an effort to find all existing studies that compared the health of these populations. The initial timeframe for our review took place between August 2017 and May 2018. We began by searching the MEDLINE/PubMed database using the keyword “Black immigrant”. Given our larger argument, we only retained studies that included a comparison with native and foreign-born Blacks. We excluded studies that only included foreign-born Blacks because they do not allow for direct comparisons with native-born Blacks. This search resulted in 29 “seed studies”. We then reviewed the citations included at the end of these studies and used the “cited by” feature on Google Scholar to find studies that cited any of the seed studies. We repeated this process for each study that we found until we no longer found additional studies (i.e., checking the references at the end of studies for more studies and finding all known citations of each study that we identified, using Google Scholar). This process identified 139 studies. We then re-ran this process again in April 2022. We reviewed all the 139 previous identified studies and used the “cited by” feature of Google Scholar to identify recently published studies that cited any of our 139 previously identified studies. As we identified more recently published studies, we then checked their references for previously unidentified studies. This process resulted in a total of 208 studies of health disparities between native and foreign-born Blacks. As the focus was on health outcomes, we excluded studies that focused on health-seeking behaviors and the use of health services. While some studies also controlled for health status co-morbidity, we did not specify this in our tables, as health status did not fit within our set of principal causes for population health.

## 5. Results

**Figure 1:**
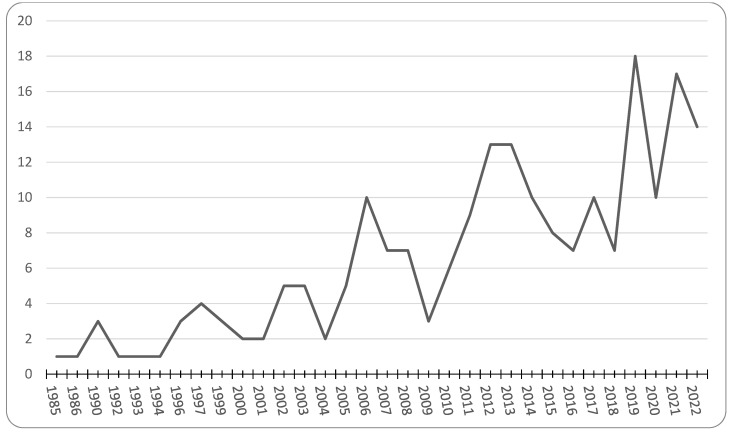
Number of Publications Comparing native- and foreign-born Blacks, By Year.

**Figure 2:**
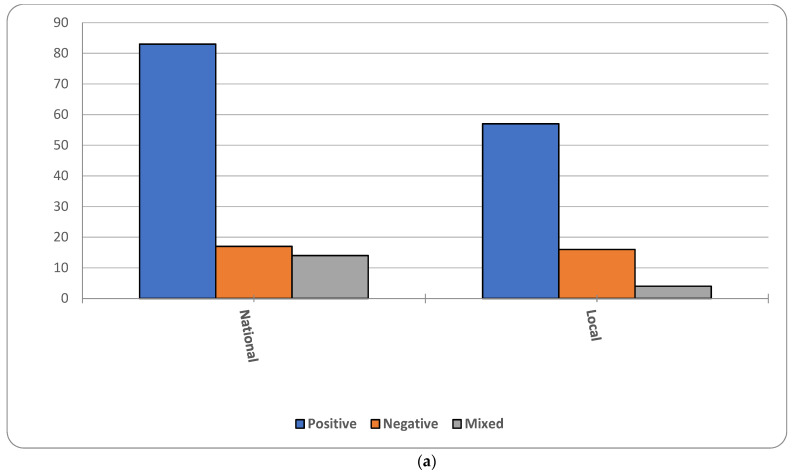

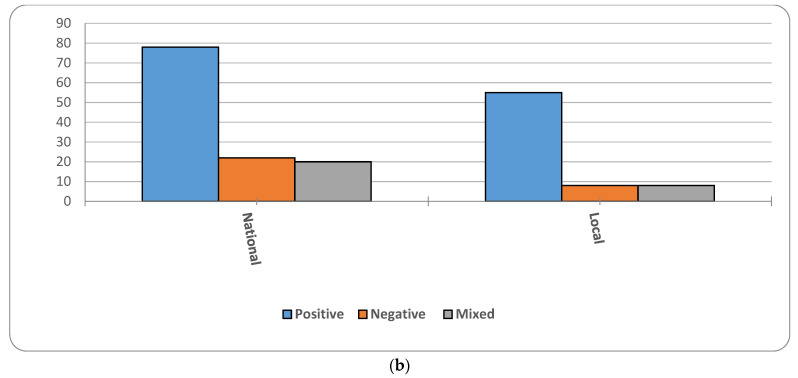
(**a**); Proportion of studies affirming hypothesis, Bivariate results (**b**) Proportion of studies affirming hypothesis, Multivariate results.

We organized the studies that we identified in Table 1 according to the Institutes of the National Institutes of Health. Most of the studies controlled for differences in socioeconomic status (194), followed by environment (55) and health behaviors (60), while very few considered genetics (6). The majority of national studies used data from the National Survey of American Life (NSAL, 45; [79]), followed by the National Health Interview Survey (NHIS, 34), the National Health and Nutrition Examination Study (NHANES, 11) and the U.S. Census (10). The remaining studies included small non-probability samples from specific community locations. Figure 1 shows the overall trend in the number of studies. The first study appeared in 1985. Since then, there has been a general increase in the rate of publications, but this increase has tapered off in recent years. Figure 2a,b shows that most studies, both bi-variate (or descriptive) and multivariate (or adjusted), report better health among foreign-born Blacks. We use the term “positive” to infer that our basic hypothesis holds true in these cases—i.e., foreign-born Blacks have better health than native-born Blacks. We use the term “negative” to denote the opposite and “mixed” when multiple outcomes had different “directions.” Although these populations are sometimes different on measures of health behavior, socioeconomic status and environment, our summary of the multivariate figures suggests that studies that account for these factors often do not provide a full accounting for health disparities between these populations.

## 6. Mental Health

According to Table 1, the most often studied set of health outcomes falls under the scope of mental health. A total of 40 studies examined differences in mental health between native and foreign-born Blacks. Studies that assessed mental health disorders used either clinical assessments or validated self-report scales. A total of 26 studies used clinical, structured interviews that diagnosed the respondent using classifications in the Diagnostic and Statistical Manual of Mental Health Disorders, Fourth Edition (DSM-IV). Some studies that used DSM-IV classifications also used the World Mental Health Composite International Diagnostic Interview (WMH-CIDI). Cohen and colleagues [80] used clinically diagnosed mental health disorders assessed by medical chart reviews. A few studies used The Center for Epidemiologic Studies Depression Scale (7, CES-D), all of which used the NSAL. The remaining studies used validated self-report scales evaluating self-rated mental health and the Short Form–8 or 12 scales.

These studies present a mixed picture across both national and local community samples and within and across mental health categories. Most of the studies used nationally representative samples (37 of 40), of which 34 of the 37 used data from the NSAL. Studies using the NSAL evaluated a range of mental health outcomes including, but not limited to, depressive symptomatology, mania, panic disorders, phobias, anxiety disorders, suicidality and developmental disorders. Even when accounting for the variability across mental health outcomes, there was no clear pattern in mental health disparities between native and foreign-born Blacks. For example, the most common mental health outcome, depressive symptomatology, had studies that varied in results, with most studies either having better or the same depressive symptoms for these populations. This pattern was also evident in the three local studies, which took place in a range of metropolitan areas in the East and Midwest [81,101,242].

## 7. Maternal and Child Health

We identified 42 maternal and child health studies in Table 1, making this the most studied physical health outcome. Of these 42 studies, 26 focused on low birthweight (LBW) and/or preterm birth (PTB). These studies mostly used vital records data, one study used census data, another study used data from the National Center of Health Statistics, and two studies relied on local samples. The study that used data from the U.S. Census [130], found a relatively equal rate of adverse birth outcomes between native and foreign-born Blacks. The other 14 national and local studies consistently found that foreign-born Blacks had lower rates of adverse birth outcomes than native-born Blacks.

Studies that examined low birthweight, defined as an infant born under 2500 g, found that native-born Blacks had higher rates of infants born at a low birthweight than foreign-born Blacks [69,118,120,121,123,124,125,126,127,128]. For instance, a study using vital statistics data [126] found that foreign-born Blacks had a reduced risk of giving birth to a low birthweight infant by approximately 25% when compared to native-born Blacks.

Studies that examined preterm birth, defined as an infant born prior to 37 weeks of gestation, found that native-born Blacks had higher rates of infants born preterm than foreign-born Blacks [18,69,128,129,131]. A recent study that used birth record data from 27 states [131] found that, compared to native-born Blacks, foreign-born Blacks had significantly lower rates of PTB. When examining the foreign-born Blacks by region of origin, this study also found that Sub-Saharan African-born Black women had significantly lower rates of PTB compared to Caribbean-born Black women.

## 8. Cardiovascular

In Table 1, we list 26 studies of disparities in cardiovascular health between native and foreign-born Black populations. National studies consistently show higher blood pressure and a greater risk of hypertension among native-born Blacks, but several local studies paint a more complex picture. Nationally, about 37% of native-born Blacks report hypertension diagnosis, compared to a little over 20% of foreign-born Blacks [171,173]. Although much less common, several studies consider other indicators of cardiovascular health, including heart trouble and problems with blood circulation. Native-born Blacks appear to have a higher rate of death by coronary heart disease than Blacks born in the Caribbean [157]. Foreign-born Black women have lower rates of myocardial infraction than native-born Black women [161], and a small study of 125 native-born and 150 Caribbean-born Black hospital patients in New York City found that the native-born were more likely to have suffered a myocardial infraction, but no differences in previous cerebrovascular disease or peripheral vascular disease [166]. Another study of the 1988–1994 NHANES III shows that foreign-born Blacks had a lower risk of stroke and heart attack than native-born Blacks [61].

## 9. Metabolic Conditions

Metabolic conditions include diabetes mellitus, abnormal cholesterol levels and potential complications with obesity. In Table 1, we identified 21 of these studies. Studies measured obesity using self-reported height and weight. Nine out of ten studies reported that native-born Black adults had higher obesity than foreign-born Black adults [170,187,188,189,190,191,194,251]. Most studies evaluated the NHIS or NHANES. The picture for diabetes was generally mixed [22,161,173,179,180,181,182]. Like obesity-related studies, national studies used NHIS or NHANES. National studies more often showed better outcomes for foreign-born Blacks. The three local studies reported similar trends between the two groups. The only study that reported better diabetes outcomes among native-born Blacks assessed type 1 diabetes (T1D) among youth in Washington state, showing that foreign-born Black populations had T1D prevalence rates that were four times higher than African American youth (6.20/1000 vs. 1.56/1000; [181]).

## 10. Cancer

We list 30 cancer studies in Table 1. The majority were local and primarily conducted in the Northeast [196,197,199,200,207]. While cancer encompasses a range of outcomes, the outcomes evaluated were specific to the reproductive system (e.g., breast, cervical, and the human papillomavirus). The national studies included data from the NHIS, NHANES and the U.S. Census [58,202,203,205]. The outcomes focused on cancer-related behaviors such as prostate cancer screening, pap smear tests and tobacco use. These studies presented mixed findings. Seven of the 17 local studies reported that foreign-born Blacks had worse cancer outcomes than native-born Blacks, and the other studies were generally mixed. The most recent of these studies found that native-born Black women reported that HPV vaccinations were more accessible to them than foreign-born Black women (Χ = 4.19, p < 0.05; [207]). Eight of the 12 national studies reported that foreign-born Blacks had better cancer outcomes and cancer-related behaviors than native-born Blacks. Some studies that reported that native-born Black populations had better cancer-related outcomes related to cancer screenings and outcomes ([(OR=3.37, 95% CI (1.89, 5.96)]; [205]).

## 11. Substance Use and Alcohol Use

In Table 1, twenty-four studies focused on substance and alcohol use outcomes. Substance use disorders occur when recurrent use of alcohol and/or drugs clinically impedes personal health [252]. The 16 studies on substance use disorders evaluated marijuana use, tobacco use and use of controlled substances. Using the National Longitudinal Study of Adolescent to Adult Health (or Add Health), Bui and colleagues, 2013 [232] assessed substance use disorders in Black adolescents and found that there were no differences in substance use when comparing native and foreign-born Blacks. The remaining national studies reported that, among adults, foreign-born Blacks were less likely to report substance use disorders than native-born Blacks. The three studies that were conducted in local settings, in Northeastern cities, were consistent with the national studies [118,231].

Eight of the 24 studies focused on alcohol use disorders. Alcohol use encompassed studies that evaluated binge drinking, alcohol abuse and alcohol use initiation. These studies trended towards better outcomes for foreign-born Blacks. Three studies deviated from this general pattern. A local study that evaluated alcohol initiation in Black adolescents found that Caribbean-born adolescents were more likely to have been in contact with alcohol than the native-born adolescents ([OR = 1.51; 95%CI (1.18–1.95)]; [226]). An examination of the National Epidemiological Survey on Alcohol and Related Conditions (NESARC) assessed alcohol use in the past 12 months and risky alcohol use behaviors (i.e., binge drinking) and found that, before adjusting for relevant covariates, foreign-born Blacks were less likely than native-born Blacks to drink or drink in a risky manner [229]. However, the adjusted estimates showed that foreign-born Black women were more likely to report using alcohol in the past 12 months than native-born women [229]. Overall, substance and alcohol use disorders are more likely among native-born than foreign-born Black adults; however, this pattern may not hold for adolescents.

## 12. Health-Related Quality of Life

We list 23 studies related to health-related quality of life in Table 1. For our review, health-related quality of life studies included self-rated physical and mental health, aging, as well as epidemiological studies assessing morbidity and mortality. Most of the studies evaluated national data, with the majority being from the NHIS (8), U.S. Census (7), and the NSAL (5). Two studies focused on local populations in Boston, Massachusetts and Florida [242,253]. When compared to foreign-born Blacks, all studies found that native-born Blacks reported poorer physical and/or mental health.

## 13. Discussion

We have joined others in offering the comparative study of native and foreign-born Blacks as a useful approach to the study of the social determinants of population health disparities and for improving our understanding of the complex interplay between social context and population characteristics in various health outcomes [12,13,14,15,16,17]. Our assessment is that, while disparities in mental health are complex, across a variety of physical health outcomes, on average, foreign-born Black populations have better health profiles than native-born Black populations—before and after controlling for various measures of health behaviors, socioeconomic status, environmental factors and/or genomics. As very few studies have been able to account for health differences between these populations, there is much more for us to learn. We conclude with some preliminary thoughts on the trends we reviewed, what we believe to be important omissions and promising opportunities for largely descriptive research in the near term, and how studies might use this comparative research design to advance our understanding of the social determinants of (racialized) population health over the longer term.

Most of the studies we reviewed are on mental health outcomes; however, the overall pattern of findings in this area is less clear than the pattern for physical health. There might be a range of explanations for this pattern. One thing to consider is that this is an artifact of measurement bias. Not only is mental health more difficult than physical health to assess in survey studies, but our current measures may not be culturally equivalent for these populations [254,255]. Conversely, measures of physical health outcomes might be more valid and reliable across these populations. That said, it is also possible that existing studies have revealed the true underlying pattern; however, mental health is complex, and so, for different dimensions of mental health, disparities between native and foreign-born Blacks are different. Therefore, we believe that, in addition to paying greater attention to disparities within a given mental health outcome, we need more studies that assess the relative construct validity of mental health measures across these populations.

We also observed a few areas of research that could benefit from more attention. For example, there are very few studies of cancer disparities. Most of the work on cancer draws on small local studies and involves screening for the presence of disease. We also need more studies that include the children and grandchildren of foreign-born Blacks. Exceedingly few studies considered the role of time since migration or the ways in which return migration might shape these disparities. While some have likely avoided an assessment of years since migration because of concerns about model identification, studies from the labor market show that these models are “identified” [34,44]. Another strategy is to estimate separate models, parsing foreign-born Blacks by different groups of years since migration (e.g., <1 to 5, >5 to 10, etc.). Finally, we only found two studies that considered any genetic differences between these populations [38,74].

In addition to a more refined description of health disparities between native and foreign-born Blacks, we continue to believe that the comparative study of these populations can improve our larger understanding of the social determinants of (racialized) disparities in population health. First, since these populations are both racially classified as Black in the U.S., sharing a multivariate distribution of skin color, hair texture and craniofacial bone structure and very likely have similar distributions of African genetic ancestry, health disparities between these populations are not likely to invoke explanations that, knowingly or unknowingly, promote the notion that race is a biological or genetic construct. That is, when or if social factors (e.g., health behaviors, socioeconomic status, and environmental factors) do not account for health disparities between native and foreign-born Blacks, any speculation on unobserved genetic differences will not be mired in contentious debates on the genetic basis of racial classification. Indeed, our review shows that several studies considered various social factors but that there remained a residual health difference between native and foreign-born Blacks. We cannot attribute this residual difference to differences in racialized notions of population genetics. This restriction will improve clarity on the nature of racialized differences in population health by muting such speculation.

The comparative study of these populations might also provide greater insight into the links between racial discrimination and health. While native and foreign-born Blacks both report experiences with racial discrimination, they may interpret or process these experiences differently. Differences in perception include differences in whether an act constitutes racial discrimination and differences in the meaning or importance of the experience. While these differences are also present among native-born Blacks, there are potentially unique reasons for similar differences between native and foreign-born Blacks. For instance, while any given two native-born Blacks might disagree on whether an experience was racially discriminatory or may differ on the importance of their experience, similar differences in perception between native and foreign-born Blacks might be uniquely motivated by nativity or differences in orientation to the centrality of American slavery in their life histories. As foreign-born Blacks are not decedents of the American “colonial situation” of chattel slavery [29] and are largely in the country by choice, except for instances of forced migration (and often with previous knowledge of American racism), their interpretation of the event might resonate differently than for native-born Blacks. While the former might understand the experience as an inconvenient nuisance in their immediate circumstance, for the latter, the same experience might trigger psycho-historical traumas that are rooted in the belief that this experience is a continuance of generations of dehumanization and restricted freedoms. While foreign-born Blacks often hail from countries with histories of slavery and colonialism, foreign-born Blacks might experience or read anti-Black and/or racialized experiences in the United States as existing outside of or unrelated to histories of slavery and colonialism in their sending countries, resulting in a different level or kind of “dose response”. Therefore, studies that compare these stress processes might help us to better understand the psychosocial factors that help link historical traumas to health outcomes [77,78].

## 14. Conclusions

Finally, there is the issue of the collection and availability of nationally representative data on these populations. For the first time, between 2001 and 2004, the Program for Research on Black Americans at the Institute for Social Research at the University of Michigan at Ann Arbor collected nationally representative data on both native-born Blacks and Afro-Caribbeans (including known *f* probabilities; [16,79]). While researchers have found this dataset tremendously fruitful (as shown above), the dataset is aging (very well), but it does not include a representative sample of foreign-born Blacks from African or other countries. It is also cross-sectional and does not include biomarkers for health and genomics. As we approach the 20th anniversary of this novel and productive data collection, it may be time to begin the process of planning the next major national data collection with the aim of understanding “Race, Ethnicity and the African Diaspora in the United States”—one that might take the best from other national data collections, such as the National Survey of Black Americans, the National Survey of American Life, the National Longitudinal Study of Adolescent to Adult Health and the Health and Retirement Survey.

## Figures and Tables

**Table 1:** Stress, behavior and outcomes.

	Author, Year	Study Population (N)	Sample	Measured Outcome	Bivariate	Multivariate
Mental health	Cohen, 1997 [80]	African American adults (135) Caribbean-born Black adults (91)	New York City, NY	Mental health disorder: Medical chart review	Negative	Negative
Mental health	Miranda, 2005 [81]	African American women (7965) African-born Black women (913) Caribbean-born Black women (273)	Washington, DC	Depressive symptoms: Prime-MD assessed	Positive	Positive
Mental health	Joe, 2006 [82]	African American adults (3570) Black Caribbean adults (1621)	National (NSAL)	Lifetime suicide ideation: self-reported	Negative	Mixed
Mental health	Jackson, 2007 [83]	African American adults (3570) Black Caribbean adults (1621)	National (NSAL)	Mental disorders (DSM-IV): WMH-CIDI assessed	Negative	Negative
Mental health	Williams, 2007 [84]	African American adults (3570) Black Caribbean adults (1621)	National (NSAL)	Major depressive disorder (DSM-IV): WMH-CIDI assessed	Positive	Negative
Mental health	Williams, 2007 [85]	African American adults (3570) Black Caribbean adults (1621)	National (NSAL)	Psychiatric disorders (DSM-IV): WHM-CIDI assessed	Mixed	Positive
Mental health	Taylor, 2007 [86]	African American adults (3570) Black Caribbean adults (1621) African American adolescents (810) Black Caribbean adolescents (360)	National (NSAL)	Psychiatric disorders (DSM-IV): WMH-CIDI	Not applicable	Positive
Mental health	Himle, 2008 [87]	African American adults (3570) Black Caribbean adults (1621)	National (NSAL)	Obsessive Compulsive Disorder (DSM-IV): WMH-CIDI assessed	Negative	Negative
Mental health	Himle, 2009 [88]	African American adults (3431) Black Caribbean adults (1585) Non-Hispanic white adults (6696)	National (NSAL; NCSR)	Psychiatric disorders (12-month; DSM-IV): WMH-CIDI assessed Psychiatric disorders (lifetime; DSM-IV): WHM-CIDI assessed	Negative	Negative
Mental health	Lincoln, 2010 [89]	African American adults (837) Black Caribbean adults (304)	National (NSAL)	Self-rated mental health: One-item, self-reported Depressive symptoms: CESD-12 scale	Not applicable	Positive
Mental health	Boyd, 2011 [90]	African American women (2019) Black Caribbean women (799) Non-Hispanic white women (400)	National (NSAL)	Lifetime mood disorder (DSM-IV): WHM-CIDI assessed	Mixed	Positive
Mental health	Soto, 2011 [91]	African American adults (3570) Black Caribbean adults (1438)	National (NSAL)	Generalized anxiety disorder (DSM-IV): WHM-CIDI assessed	Positive	Positive
Mental health	Taylor, 2011 [92]	African American adults (3570) Caribbean Black adults (1621)	National (NSAL)	Suicidal behavior (DSM-IV): WMH-CIDI assessed	Positive	Positive
Mental health	Aranda, 2012 [93]	African American adults (786) Caribbean Black adults (295) Non-Hispanic white adults (287)	National (NSAL)	Major depressive disorder (lifetime; DSM-IV): WMH-CIDI assessed Major depressive disorder (12-month; DSM-IV): WMH-CIDI assessed	Negative	Negative
Mental health	Doyle, 2012 [94]	African American men (1254) US-born Caribbean Black men (175) Caribbean-born Black men (458)	National (NSAL)	Mental illness (DSM-IV): WMH-CIDI assessed Mental health severity: Sheehan disability scale	Positive	Positive
Mental health	Goosby, 2012 [95]	African American adolescent-parent dyads (612) Black Caribbean adolescent-parent dyad (258)	National (NSAL)	Depressive symptoms: CESD-12 scale Adolescent stress appraisal: Cohen’s perceived scale Parent stress appraisal: one-time, self-report	Mixed	Mixed
Mental health	Ida, 2012 [96]	African American adults (2953) Caribbean Black adults (1140)	National (NSAL)	Depressive symptoms: CESD-12 scale	Positive	Positive
Mental health	Lincoln, 2010 [89]	African American adults (3570) Caribbean-born Black adults (1621)	National (NSAL)	Major depressive disorder (DSM-IV): WMH-CIDI assessed	Negative	Negative
Mental health	Lincoln, 2012 [97]	African American adults (3570) Caribbean-born Black adults (1621)	National (NSAL)	Suicide ideation: self-reported Suicide attempts: self-reported	Negative	Negative
Mental health	Woodward, 2012 [98]	African American adults (780) Caribbean Black adults (262) Latino American adults (498) Asian American adults (376) Non-Hispanic white adults (1130)	National (NSAL; NCSR, NLAAS)	Lifetime affective disorders (DSM-IV): WMH-CIDI assessed SAnxiety disorder (DSM-IV): WMH-CIDI assessed	Mixed	Negative
Mental health	Assari, 2013 [99]	African American adults (3570) Caribbean Black adults (1621) Guyanese Black adults (1142) Jamaican Black adults (1176)	National (NSAL)	Psychiatric disorders (DSM-IV): WMH-CIDI assessed SLifetime serious suicide ideation: Self-reported	Positive	Positive
Mental health	Gibbs, 2013 [100]	African American adults (7529) Caribbean Black adults (469)	National (NESARC)	Psychiatric disorders (DSM-IV): AUDADIS-IV assessed Lifetime major depressive disorder: Self-reported	Positive	Positive
Mental health	Henning-Smith, 2013 [101]	African American adults (242) Somali-born adults (288) White American adults (408)	Minnesota	Mental health: Self-reported emotional health	Positive	Positive
Mental health	Levine, 2013 [102]	African American adults (3570) Caribbean-born Black adults (1621)	National (NSAL; NCSR)	Panic disorder (lifetime; DSM-IV): WMH-CIDI assessed Panic disorder 12-month; DSM-IV): WMH-CIDI assessed	Negative	Mixed
Mental health	Marshall, 2013 [103]	African American adults (837) Caribbean Black adults (271)	National (NSAL)	Depressive symptoms: CESD-12 scale	Not applicable	Mixed
Mental health	Woodward, 2013 [104]	African American adults (1135) Caribbean black adults (426) Non-Hispanic white adults (389)	National (NSAL)	Major depressive disorder (lifetime; DSM-IV): WMH-CIDI assessed Major depressive disorder (12-month; DSM-IV): WMH-CIDI assessed	Not applicable	Mixed
Mental health	Levine, 2014 [105]	African American adults (3570) Caribbean-born Black adults (1621)	National (NSAL)	Social anxiety disorder (DSM-IV): WMH-CIDI assessed	Positive	Mixed
Mental health	Brewton-Tiayon, 2015 [106]	African American adults (3434) US-born Caribbean adults (432) Caribbean-born Black adults (1141)	National (NSAL)	Major depressive disorder (lifetime; DSM-IV): WMH-CIDI assessed Major depressive episode (lifetime; DSM-IV): WMH-CIDI assessed Depressive symptoms: CESD-12 scale	Mixed	Not applicable
Mental health	Lankarani, 2015 [107]	African American adults (396) Caribbean Black adults (131) non-Hispanic white adults (75)	National (NSAL)	Major depressive disorder (lifetime; DSM-IV): WMH-CIDI assessed Major depressive disorder (12-month; DSM-IV): WMH-CIDI assessed Chronic medical conditions: Self-reported	Positive	Positive
Mental health	Taylor, 2015 [108]	African American adults (3570) Caribbean-born Black adults (1621)	National (NSAL)	Major depressive disorder (lifetime; DSM-IV): WMH-CIDI assessed Major depressive disorder (12-month; DSM-IV): WMH-CIDI assessed Psychological distress: Kessler-6 scale	Mixed	Mixed
Mental health	Assari, 2016 [109]	African American adults (3570) Caribbean-born Black adults (1621)	National (NSAL)	Mental health: Self-rated	Mixed	Mixed
Mental health	Assari, 2013 [99]	African American adults (3570) Caribbean-born Black adults (1621)	National (NSAL)	Suicide ideation: Single-item self-report	Not applicable	Mixed
Mental health	Mereish, 2016 [110]	African American men (1201) Caribbean-descendent Black men (545)	National (NSAL)	Depressive symptoms: CESD-12 scale	Positive	Positive
Mental health	Molina, 2016 [111]	African American adults (3570) Caribbean-born Black adults (1418)	National (NSAL)	Major depressive disorder (12-month; DSM-IV): WMH-CIDI assessed	Mixed	Positive
Mental health	Blostein, 2017 [112]	African American adults (3570) Caribbean-born Black adults (1621)	National (NSAL)	Lifetime binge eating (DSM-IV): WMH-CIDI assessed	Not applicable	Negative
Mental health	Mouzon, 2017 [113]	African American adults (3570) Caribbean-born Black adults (1438)	National (NSAL)	Depressive symptoms: CESD-12 scale Psychological distress: Kessler-6 scale	Not applicable	Positive
Mental health	Moon, 2019 [114]	US born Black adults (1535) Foreign born Black adults (125) US and foreign-born White; Latino populations	National (NHATS)	Dementia: proxy report, AD8 score, and cognitive testing	Positive	Positive
Mental health	Erving, 2019 [115]	African Americans (1616) Caribbean Blacks (601)	National (NSAL)	Depressive symptoms: CESD-12 scale	Not applicable	Not applicable
Mental health	Ikonte, 2020 [116]	US born Black adults (10,959) Foreign born Black adults (6605) US and foreign-born White; Latino populations	National (NHIS)	Psychological distress: Kessler-6 scale	Positive	Positive
Mental health	Erving, 2021 [117]	African-American women (2084) U.S. born Afro-Caribbean women (250) Foreign-born Afro-Caribbean women (631)	National (NSAL)	Depressive symptoms: CESD-12 scale	Positive	Positive
Maternal	Cabral, 1990 [118]	African-American women (616) Foreign-born Black women (201)	Massachusetts	Intrauterine growth: Clinically derived Duration of gestation: Clinically derived Birthweight: Clinically derived	Positive	Positive
Maternal	Friedman, 1993 [119]	African-American women (n/r) Foreign-born Black women (n/r)	Massachusetts	Birthweight: Birth certificate tapes	Positive	Positive
Maternal	Wasse, 1994 [120]	African American women (526) Ethiopian-born Black women (264)	Washington State	Birthweight: Birth certificate tapes	Positive	Positive
Maternal	David, 1997 [121]	African-American women (43,322) African-born Black women (3135)	Illinois	Birthweight: Birth certificate tapes	Positive	Positive
Maternal	Hummer, 1999 [18]	African-American women (n/r) Foreign-born Black women (n/r)	National (NCHS)	Infant mortality: infant born alive survived to first birthday	Positive	Positive
Maternal	Fang, 1999 [122]	U.S. born (Southern) Black women (17,968) U.S.-born (Northeastern) Black women (155,101) African-born Black women (9362) Caribbean-born Black women (76,426) South America born Black women (11,006)	New York City, NY	Preterm Birth Risk: Birth records	Positive	Positive
Maternal	Pallotto, 2000 [123]	African-American women (67,357) Caribbean-born Black women (2265)	Illinois	Low birth weight: Clinically derived	Positive	Positive
Maternal	Collins, 2002 [124]	African-American Grandmothers (31,699) African/Caribbean-born Black Grandmothers (104)	Illinois	Low birth weight: Clinically derived	Positive	Positive
Maternal	Rosenberg, 2002 [125]	African-American women (130,681) Foreign-born Black women (72,293)	New York City, NY	Low birth weight: Clinically derived Infant mortality: State records	Positive	Positive
Maternal	Acevedo-Garcia, 2005 [126]	African-American women (322,510) Foreign-born Black women (40,213)	National (Detail Natality)	Low birth weight: Clinically derived	Positive	Positive
Maternal	Howard, 2006 [69]	African-American women (88,966) West-Indian/Brazilian Black women (47,050) South/Central American Black women (15,234) African-born Black women (10,875) Puerto Rican Black women (3948) European Black women (1028) Asian Black women (747) Cuban Black women (191)	New York City, NY	Low birth weight: Clinically derived Preterm delivery: Clinically derived	Positive	Positive
Maternal	Grady, 2007 [127]	African-American women (17,938) Foreign-born Black women (18,459)	New York City, NY	Birthweight risk: Geocoded	Positive	Positive
Maternal	Dominguez, 2009 [128]	African-American women (185) Foreign-born Black women (114)	Boston, MA	Preterm delivery: Clinically derived Low birthweight: Clinically derived Infant mortality: Clinically derived	Positive	Positive
Maternal	Elo, 2010 [60]	African-American Pregnant women (2816) African-Born Pregnant women (106) Caribbean-Born Pregnant women (179)	Philadelphia, PA	Smoking: Self-report, 12 months prior to pregnancy Alcohol use: Self-report, 12 months prior to pregnancy Marijuana use: Seld-report, 12 months prior to pregnancy	Positive	Positive
Maternal	Mason, 2010 [129]	African-American women (141,969) Caribbean-born Black women (87,026) African-born Black women (21,088)	New York City, NY	Preterm Birth Risk: Birth records	Positive	Positive
Maternal	Bloch, 2011 [130]	African-American women (24,165) Foreign-born Black women (6136)	Philadelphia, PA	Preterm delivery rate: Geospatial	Negative	Negative
Maternal	Elo, 2014 [131]	African-American women (296,787) Foreign-born Black women (47,334)	27 States	Rates of Prematurity: Clinically estimated gestational age (20–37 weeks) Small for Gestational Age: weight <10th percentile given gestational week	Positive	Positive
Maternal	Hendi, 2015 [132]	African American women (34,371) African-born women (1416) Caribbean-born women (3151)	National (NHIS)	Childhood health: self-report	Positive	Positive
Maternal	DeSisto, 2018 [133]	US born Black women (337,141) Foreign born Black women (63,493)	National (Detail Natality)	Preterm Birth Risk: Birth records	Positive	Positive
Maternal	Singh, 2018 [19]	US born Black women (1,004,997) Foreign born Black women (177,299) US and foreign-born White; Asian populations	National (Detail Natality)	Maternal Hypertension Risk: Birth records	Positive	Positive
Maternal	Oliver, 2018 [134]	US born Black women (303,028) African born Black women (10,966) Somalia-born women (8480)	Ohio	Preterm delivery rate: Clinically derived	Mixed	Mixed
Maternal	Kirby, 2019 [135]	US born Black women (1,561,600) Foreign born Black women (254,052) US and foreign-born White; Latino populations	National (Detail Natality)	Birth Defect Risk: Birth records	Positive	Positive
Maternal	Elsayed, 2019 [136]	US born Black women (340) Foreign born Black women (107)	Newark, NJ	Preterm Birth Risk: Clinically derived	Positive	Positive
Maternal	Singh, 2019 [137]	US born Black women (n/r) Foreign born Black women (n/r) US and foreign-born White; Asian; Latino populations	National (Detail Natality)	Pre-pregnancy Obesity: Self-reported	Positive	Positive
Maternal	Scott, 2020 [138]	US born Black women (7222) Foreign born Black women (1387)	California	Gestational Diabetes Risk: Clinically derived Preterm Birth Risk: Clinically derived	Positive	Positive
Maternal	Araneta, 2020 [139]	US born Black women (6673) Foreign born Black women (2083) US and foreign-born White; Asian; Latino populations	San Diego, CA	Preterm delivery: Clinically derived	Positive	Positive
Maternal	Hoyt, 2020 [140]	US born Black women (1101) Foreign born Black women (151)	National (NBDPS)	Birth Defect Risk: Birth records	Positive	Positive
Maternal	Boakye, 2021 [141]	US born Black women (1607) Foreign born Black women (1092) US and foreign-born White; Latino populations	Boston, MA	Preeclampsia Risk: Clinically derived	Positive	Positive
Maternal	Hong, 2021 [142]	African-American (275) Foreign born Haitian women (151)	Boston, MA	Preeclampsia Risk: Clinically derived	Negative	Positive
Maternal	McKenzie-Sampson, 2021 [143]	US born Black women (129,775) Foreign born Black women (16,896)	California	Preterm Birth Risk: Clinically derived	Positive	Positive
Maternal	Adegoke, 2021 [144]	US born Black women (1722) Foreign born Black women (2994) US and foreign-born White; Latino populations	Boston, MA	Preterm Birth Risk: Clinically derived Hypertensive disorders: Clinically derived Low birthweight: Clinically derived Intrauterine fetal demise: Clinically derived	Positive	Positive
Maternal	Boakye, 2021 [141]	U.S. born Black women (1605) Foreign born Black women (1093) US and foreign-born White; Latino populations	Boston, MA	Preeclampsia Risk: Clinically derived	Positive	Positive
Maternal	Andrasfay, 2021 [145]	US born Black women (47,324) Foreign born Black women (1157) US and foreign-born White; Asian; Latino populations	California	Low birthweight (less than 2500 g): Clinically derived Preterm Birth Risk: Clinically derived	Positive	Positive
Maternal	Green, 2021 [146]	US born Black women (n/r) Foreign born Black women (n/r) US and foreign-born White; Asian; Latino populations	National (ECLS-B)	Pregnancy-related obesity: Self-reported	Positive	Mixed
Maternal	Park, 2021 [147]	US born Black women (1849) Foreign born Black women (1849) US and foreign-born White; Latino populations	Boston, MA	Tobacco use: Self-reported Alcohol use: Self-reported Postnatal CMDs: Self-reported	Positive	Positive
Maternal	Blebu, 2021 [148]	US born Black women (83,169) African born Black women (7151) Caribbean-born Black women (943)	California	Preterm delivery: Clinically derived	Positive	Mixed
Maternal	Kwapong, 2022 [149]	US born Black women (1607) Foreign born Black women (1092) US and foreign-born White; Latino populations	Boston, MA	Preterm delivery: Clinically derived	Positive	Positive
Maternal	Minhas, 2022 [150]	US born Black women (1607) Foreign born Black women (1092)	Boston, MA	Preterm delivery: Clinically derived	Positive	Positive
Maternal	Maiyegun, 2022 [151]	US born Black women (1,746,740) Foreign born Black women (332,422) US and foreign-born White; Latino populations	National (Detail Natality)	Stillbirth Risk: Birth records	Positive	Positive
Maternal	Egbe, 2022 [152]	US born Black women (61,589) Foreign born Black women (7348) US and foreign-born White; Asian; Latino populations	Philadelphia, PA	Preterm delivery: Clinically derived	Positive	Positive
Maternal	Shah, 2022 [153]	US born Black women (1605) Foreign born Black women (1092) US and foreign-born White; Latino populations	Boston, MA	Gestational Diabetes Risk: Clinically derived	Negative	Mixed
Maternal	Belanoff, 2022 [154]	US born Black women (12,292) Foreign born Black women (14,356)	Central Massachusetts	Preterm delivery: Clinically derived	Positive	Mixed
Maternal	Noah, 2022 [155]	US born Black women (4134) Foreign born Black women (1402) US and foreign-born White; Latino populations	Houston, TX	Chlamydia, Gonorrhea, and Syphilis Risk: Clinically derived	Positive	Mixed
CVD	Fumo, 1992 [156]	African American adults (22) African-born adults (22)	Independent	Hypertension: Diurnal blood pressure	Positive	Positive
CVD	Fang, 1996 [157]	African American adults (Northern) (1,008,677) African American adults (Southern) (366,853) Black Caribbean adults (309,380)	New York City, NY	Hypertension: Mortality records	Positive	Positive
CVD	Kaufman, 1996 [158]	African American adults (1518) Caribbean Black adults (2722) African Black adults (4862)	National (ICSHB)	Hypertension: Clinical assessment Obesity: Clinical assessment	Positive	Negative
CVD	Osei, 1996 [159]	African American adults (31) African-born adults (27)	Ohio	Hypertension: Measured systolic/diastolic blood pressure	Negative	Negative
CVD	Hyman, 2000 [160]	African American adults (95) African-born adults (87)	Independent	Hypertension	Positive	Positive
CVD	Poston, 2001 [74]	African American adults (99) African-born adults (86)	Houston, TX	Hypertension: Mercury sphygmomanometer	Negative	Positive
CVD	Hicks, 2003 [161]	African American adults (Northern) (1403) African American adults (Southern) (1751) Foreign-born adults (215)	National (NHANES)	Hypertension: self-reported uncontrolled or target-organ damage	Positive	Positive
CVD	Read, 2005 [17]	African American adults (16,891) Foreign-born Black adults (2015)	National (NHIS)	Activation limitation attributed to hypertension: self-reported	Positive	Positive
CVD	Lancaster, 2006 [61]	African American adults (2062) Foreign-born Black adults (241) Foreign-born, Hispanic Black adults (4) US-born, Hispanic Black adults (21)	National (NHANES)	Coronary heart disease risk profile	Positive	Positive
CVD	Ryan, 2006 [162]	African American adults (78) Foreign-born Black adults (112)	New Hampshire	Hypertension: Measured blood pressure Physical health: Self-reported health, SF-12	Negative	Negative
CVD	Davis, 2007 [163]	African American adults (61) US-born, Black Caribbeans (62) Foreign-born Black Caribbeans (66)	South Florida	Hypertension: measured blood pressure Diabetes: fasting blood glucose Cholestrol: measured LDL	Positive	Positive
CVD	Borrell, 2008 [164]	African American adults (36,358) Foreign-born Black adults (3376)	National (NHIS)	Hypertension: self-reported doctor diagnosed	Positive	Positive
CVD	White, 2011 [165]	African American adults (2985) Foreign-born Black adults (1514)	New York City, NY	Hypertension: self-reported doctor diagnosed	Positive	Positive
CVD	Bamimore, 2012 [166]	African American adults (125) Caribbean-born Black adults (150)	New York	Myocardial infarction: self-reported Hypertension: self-reported	Negative	Not applicable
CVD	Sellers, 2012 [167]	African American adults (3570) Caribbean Black adults (445) Non-Hispanic white adults (891)	National (NSAL)	Hypertension: self-reported Obesity: BMI derived from height/weight Count of physical health problems: self-reported Self-rated health: self-reported	Negative	Negative
CVD	Yu, 2013 [168]	African American men (75) African-born men (80)	Washington, DC	Cardiometabolic diseases: self-reported (prediabetes, diabetes, insulin resistance, metabolic triad)	Mixed	Not applicable
CVD	Dagadu, 2014 [169]	African American adults (1588) Caribbean Black adults (549)	National (NSAL)	Heart trouble: Self-reported	Mixed	Mixed
CVD	O’Connor, 2014 [170]	African American adults (76) Foreign-born Black adults (138)	Washington, DC	Hypertension: measured blood pressure Type 2 diabetes: fasting glucose; 2-h glucose Visceral Adipose Tissue: Computerized tomographic scans	Negative	Negative
CVD	Brown, 2017 [171]	African American adults (4249) Foreign-born Black adults (515)	National (NHANES)	Hypertension: self-reported doctor diagnosed or measured blood pressure	Positive	Positive
CVD	Cole, 2017 [172]	African American men (817) Foreign-born Black men (310)	New York City, NY	Hypertension: measured blood pressure Hypertension awareness: self-reported hypertension	Positive	Positive
CVD	Commodore-Mensah, 2017 [173]	African American adults (40,838) African-Born Black adults (36,881) Caribbean-Born Black adults (1660)	National (NHIS)	Hypertension: self-reported doctor diagnosed	Positive	Positive
CVD	Cole, 2018 [174]	US born Black men (829) Foreign born Black men (311)	New York City, NY	Hypertension awareness: self-reported hypertension	Negative	Negative
CVD	Fang, 2018 [175]	US born Black adults (n/r) Foreign born Black adults (n/r) US and foreign-born White; Asian; Latino populations	National (NHIS)	Coronary heart disease risk profile	Not applicable	Negative
CVD	Turkson-Ocran, 2020 [176]	African-Americans (27,749) African immigrants (1345)	National (NHIS)	Coronary heart disease risk profile	Negative	Negative
CVD	Whaley, 2020 [177]	African-Americans (3570) African immigrants (1419)	National (NSAL)	Coronary heart disease risk profile	Negative	Negative
CVD	Doamekpor, 2021 [178]	US born Black adults (4693) Foreign born Black adults (2968)	National (NHANES)	Coronary heart disease risk profile	Mixed	Negative
Metabolic Conditions	Hicks, 2003 [161]	African American adults (Northern) (1403) African American adults (Southern) (1751) Foreign-born adults (215)	National (NHANES)	Type 2 Diabetes: Self-reported	Positive	Positive
Metabolic Conditions	Singh, 2006 [22]	African American adults (n/r) Foreign-born Black adults (n/r) US and foreign-born White; Asian; Latino populations	National (NHIS)	Type 2 Diabetes: Prevalence	Positive	Not applicable
Metabolic Conditions	Oza-Frank, 2013 [179]	African American adults (1179) Foreign-born Black adults (93)	Multi-site (MESA)	Type 2 Diabetes	Mixed	Not applicable
Metabolic Conditions	Ford, 2015 [180]	African American adults (42,379) African-born Black adults (1533) Latin American/Caribbean-born Black adults (3839)	National (NHIS)	Type 2 Diabetes: Self-reported, doctor diagnosed	Positive	Positive
Metabolic Conditions	O’Connor, 2015 [181]	African American Youth (53) East African Immigrant Youth (60)	Washington	Type 1 Diabetes: Medically diagnosed	Negative	Not applicable
Metabolic Conditions	Commodore-Mensah, 2017 [173]	African American adults (40,838) African-Born Black adults (36,881) Caribbean-Born Black adults (1660)	National (NHIS)	Hypertension: self-reported doctor diagnosed	Positive	Positive
Metabolic Conditions	Harvey, 2017 [182]	African American women (10) Caribbean-born women in US (8) Caribbean-born women in US Virgin Islands (24)	Connecticut & US Virgin Islands	Diabetes self-management behaviors: self-reported diet, physical activity, medication adherence, foot care	Positive	Not applicable
Metabolic Conditions	Horlyck-Romanovsky, 2019 [183]	US born Black adults (6297) Foreign born Black adults (3701)	New York	Type 2 Diabetes: Prevalence	Mixed	Mixed
Metabolic Conditions	Engelman, 2019 [184]	US born Black adults (62381) Foreign born Black adults (6444) US and foreign-born White; Asian; Latino populations	National (NHIS)	Type 2 Diabetes: Prevalence	Positive	Mixed
Metabolic Conditions	Ogunwole, 2022 [185]	U.S. born NH Black adults Foreign born NH Black adults US and foreign-born White; Asian; Latino populations	National (NHIS)	Gestational diabetes mellitus	Negative	Mixed
Metabolic Conditions	Choi, 2022 [186]	US born Black adults (n/r) Foreign born Black adults (n/r) US and foreign-born White; Asian; Latino populations	National (NHANES)	Type 2 Diabetes: Prevalence	Negative	Negative
Metabolic Conditions	Antecol, 2006 [187]	African American adults (35,642) Foreign-born Black adults (2446) US and foreign-born White; Latino populations	National (NHIS)	Obesity: BMI derived from height/weight	Positive	Positive
Metabolic Conditions	Bennett, 2007 [188]	African American adults (394) Foreign-born Black adults (157)	Massachusetts	Obesity: BMI derived from height/weight	Positive	Positive
Metabolic Conditions	Sanchez-Vaznaugh, 2008 [189]	African American adults (1835) Foreign-born Black adults (106) US and foreign-born White; Asian; Latino populations	California	Obesity: BMI derived from height/weight	Positive	Positive
Metabolic Conditions	Barrington, 2010 [190]	African American adults (n/r) Foreign-born Black adults (n/r) US and foreign-born White; Latino populations	National (NHIS)	Obesity: BMI derived from height/weight	Positive	Positive
Metabolic Conditions	Wen, 2013 [191]	African American adults (952) Foreign-born Black adults (102)	National (NHANES)	BMI: Clinically measured Abdominal obesity: Calculated waist circumference (≥102 cm)	Positive	Positive
Metabolic Conditions	Assari, 2014 [192]	African American adults (3570) Caribbean Black adults (1621)	National (NSAL)	Obesity: BMI derived from height/weight	Not applicable	Mixed
Metabolic Conditions	O’Connor, 2014 [170]	African American adults (76) African-born Black adults (138)	Washington, DC	BMI: Calculated Waist Circumference: measured Subcutaneous adipose tissue: Computerized tomographic scans	Positive	Positive
Metabolic Conditions	Sullivan, 2014 [193]	African American adults (3570) Caribbean Black adults (1621) Non-Hispanic white (891)	National (NSAL)	Obesity: BMI derived from height/weight	Not applicable	Positive
Metabolic Conditions	Mehta, 2015 [194]	African American adults (33,771) African-born Black adults (1435) Latin American/Caribbean-born Black adults (2520)	National (NHIS)	Obesity: BMI derived from height/weight	Positive	Positive
Metabolic Conditions	Cuevas, 2019 [195]	US born Black adults (n/r) Foreign born Black adults (n/r) US and foreign-born White; Asian; Latino populations	National (NESARC-III)	Obesity: BMI derived from height/weight	Positive	Positive
Cancer	Fruchter, 1985 [196]	African-American women (237) English, Caribbean-born women (227) Spanish, Caribbean-born women (70) Haitian-Born women (361)	New York City, NY	Cancer screening: Breast, Cervical	Negative	Not applicable
Cancer	Fruchter, 1986 [197]	African-American women (1477) English, Caribbean-born women (256) Haitian-Born women (121)	New York City, NY	Cancer screening: Cervical	Positive	Not applicable
Cancer	Fang, 1997 [157]	U.S. born Black adults (Southern) (366,853) U.S. born Black adults (Northeastern) (1,008,677) Caribbean-born Black adults (309,380)	New York City, NY	Cancer survival: All sites	Not applicable	Mixed
Cancer	Magnus, 2004 [198]	African-American men (56) Caribbean-American men (29) Haitian-American men (11) African-born Black men (4)	Southern Florida	Cancer screening: Prostate	Not applicable	Not applicable
Cancer	Garbers, 2006 [199]	African-American women (148) Caribbean-Born women (146)	New York City, NY	Cancer screening: Breast	Negative	Negative
Cancer	Bennett, 2008 [200]	African American men (447) Foreign-born Black men (218)	Boston, MA	Tobacco use: Self-reported smoking status	Not applicable	Positive
Cancer	Taioli, 2010 [201]	African American women (593) Trinidad and Tobago women (2618) Guyana (499)	New York, NY, Trinidad and Tobago; Guyana	Cancer survival: Breast	Negative	Negative
Cancer	Odedina, 2011 [202]	African-American men (2405) African-Born Black men (315) Caribbean-Born Black men (320)	National (NHANES)	Cancer screening: Prostate	Positive	Positive
Cancer	Wade, 2013 [203]	African American adults (4253) Foreign-born Black adults (460)	National (US Census)	Tobacco use: Self-reported smoking status	Not applicable	Positive
Cancer	Consedine, 2014 [204]	Black Caribbean Descendent adults (n/a) Caribbean-Born Black adults (n/a)	Not available	Cancer screening: Breast, Prostate, Colorectal, Cervical	Negative	Not applicable
Cancer	Forney-Gorman, 2016 [205]	African-American women (620) African-Born Black women (36)	National (NHIS)	Cancer screening: Pap smear test	Negative	Negative
Cancer	Pinheiro, 2016 [206]	U.S. born Black adults (16,119) Foreign-born Black adults (4113)	Southern Florida	Cancer survival: All sites	Not applicable	Positive
Cancer	Ashing, 2017 [207]	African American Black women (129) Foreign-born Black women (53) US-born Latina women (57) Foreign-born Latina women (144)	Southern California	HPV vaccine safety: self-reported HPV vaccine efficacy: self-reported	Negative	Not applicable
Cancer	Barreto-Coelho, 2019 [208]	African American adults (507) US-born Caribbean Black adults (624)	Southern Florida	Cancer survival: Breast	Positive	Positive
Cancer	Hallowell, 2019 [209]	US born Black adults (7172) Foreign born Black adults (591) US and foreign-born White; Asian; Latino populations	National (US Census)	Cancer survival: Cervical	Positive	Positive
Cancer	Schlumbrecht, 2019 [210]	African American adults (105) Caribbean Black adults (90)	Southern Florida	Cancer survival: Endometrial	Positive	Positive
Cancer	Cofie, 2019 [211]	US born Black women (140,670) Foreign born Black women (14,837) US and foreign-born White; Asian; Latino populations	National (NHIS)	Cancer screening: Breast	Positive	Positive
Cancer	Bhattacharya, 2019 [212]	US born Black adults (621) Foreign born Black adults (81) US and foreign-born White; Latino populations	National (NHANES)	HPV infection	Positive	Positive
Cancer	Boakye, 2019 [213]	US born Black men (1319) Foreign born Black men (204) US and foreign-born White; Latino populations	National (NHIS)	HPV vaccine safety: self-reported HPV vaccine efficacy: self-reported	Negative	Negative
Cancer	Endeshaw, 2019 [214]	US born Black men (17,712) Foreign born Black men (1104) US and foreign-born White; Asian; Latino populations	National (NCHS)	Cancer survival: Liver	Positive	Positive
Cancer	Hallowell, 2019 [215]	US born Black adults (310,684) Foreign born Black adults (15,788) US and foreign-born White; Asian; Latino populations	National (NCHS)	Cancer survival: All sites	Positive	Positive
Cancer	Hallowell, 2019 [216]	US born Black men (10,431) Foreign born Black men (878) US born Black women (7188) Foreign born Black women (684) US and foreign-born White; Asian; Latino populations	National (NCHS)	Cancer survival: Gastric	Positive	Positive
Cancer	Pinheiro, 2020 [217]	African-American adults (7,350,702) Afro-Caribbean adults (1,227,555) African adults (372,082)	California, Florida, Minnesota and New York	Cancer survival: All sites	Positive	Positive
Cancer	Amuta-Jimenez, 2020 [218]	African American women (335) Black immigrant women (115)	National (NHIS)	Cancer screening: Cervical	Positive	Positive
Cancer	Donley, 2020 [219]	U.S.-born and foreign-born women aged 21–74 years; Black/African-American, European, Asian/Pacific Islander, and other Hispanic/Latino.	Independent	Cancer screening: Cervical and Breast	Positive	Positive
Cancer	McRoy, 2021 [220]	US born Black adults (4544) Foreign born Black adults (572)	National (NHANES)	Cancer survival: All sites	Negative	Negative
Cancer	Blackman, 2021 [221]	US born Black adults (208) Caribbean born Black adults (103) African-born Black adults (46)	Philadelphia, PA	Cancer screening: Colorectal	Positive	Positive
Cancer	McElfish, 2021 [222]	US born Black adults (n/a) Foreign born Black adults (n/a) US and foreign-born White; Asian; Latino populations	National (NHIS)	HPV vaccine safety: self-reported	Negative	Negative
Cancer	Pinheiro, 2021 [223]	US born Black adults (3568) Caribbean Black adults (1381) US and foreign-born White; Latino populations	Florida, New York	Cancer survival: Endometrial	Positive	Positive
Cancer	Llanos, 2022 [224]	US born Black adults (38,834) Foreign born Black adults (5433)	New Jersey	Cancer survival: All sites	Positive	Positive
Cancer	Millender, 2022 [225]	US born Black adults (n/a) Foreign born Black adults (n/a) US and foreign-born White; Latino populations	Independent	Cancer screening: Prostate	Positive	Positive
Alcohol	Cabral, 1990 [118]	African American women (616) Foreign-born Black women (201)	Boston, MA	Alcohol use during pregnancy: Self-reported	Positive	Positive
Alcohol	Cohen, 1997 [80]	African American adults (135) Caribbean-born Black adults (91)	New York City, NY	Alcohol dependence: Medical chart review	Positive	Positive
Alcohol	Epstein, 2002 [226]	African American adolescents (2281) Caribbean-born Black adolescents (931)	New York City, NY	Alcohol use: Self-reported frequency	Positive	Mixed
Alcohol	Lucas, 2003 [59]	African American men (13,921) Foreign-born Black men (1486)	National (NHIS)	Alcohol use: Self-reported quantity/frequency past 12 months	Positive	Positive
Alcohol	Hunte, 2012 [227]	African American adults (3917) Foreign-born Black adults (1091)	National (NSAL)	Alcohol use disorder (DSM-IV): WMH-CIDI assessed	Positive	Positive
Alcohol	Lo, 2012 [228]	African American adults (2110) Foreign-born Black adults (193)	National (NHIS)	Binge drinking: Days in past year consumed 5 drinks/day Quantity of alcohol consumed: Average # drinks consumed/day	Mixed	Mixed
Alcohol	Gibbs, 2013 [100]	African American adults (7529) Caribbean Black adults (469)	National (NESARC)	Alcohol use (DSM-IV): AUDADIS-IV assessed	Positive	Positive
Alcohol	Szaflarki, 2017 [229]	African American adults (3969) Foreign-born adults (319)	National (NESARC)	Alcohol use disorder: DSM-IV assessed	Positive	Negative
Substance Use	Cabral, 1990 [118]	African American women (616) Foreign-born Black women (201)	Boston, MA	Cigarette use: Self-reported Marijuana use: Self-reported Cocaine use: Self-reported Opiate use: Self-reported	Positive	Positive
Substance Use	King, 1999 [58]	African American adults (15,660) Foreign-born Black adults (1078)	National (NHIS)	Smoking status: Self-reported	Positive	Positive
Substance Use	Lucas, 2003 [59]	African American adults (13,921) Foreign-Born Black adults (1486)	National (NHIS)	Smoking: Self-reported status	Positive	Positive
Substance Use	Broman, 2008 [230]	African American adults (3570) Black Caribbean adults (1621)	National (NSAL)	Substance abuse (DSM-IV): WMH-CIDI assessed Substance dependence (DSM-IV): WMH-CIDI assessed	Positive	Positive
Substance Use	Hoffman, 2008 [231]	African American adults (300) West Indian-born Black adults (287)	New York City, NY	Substance use: AUDIT scale	Positive	Positive
Substance Use	Bui, 2012 [232]	African American adolescents (3828) Foreign-born Black adolescents (75)	National (Add Health)	Tobacco use: use 30 days prior; yes/no Marijuana use: use 30 days prior; yes/no	Mixed	Mixed
Substance Use	Gibbs, 2013 [100]	African American adults (7529) Caribbean Black adults (469)	National (NESARC)	Substance use (DSM-IV): AUDADIS-IV assessed	Positive	Positive
Substance Use	Lacey, 2016 [233]	African American adults (3570) Caribbean Black adults (1621) Guyanese Black adults (1142) Jamaican Black adults (1176)	National (NSAL); Guyana; Jamaica	Substance use (DSM-IV): WMH-CIDI assessed	Positive	Positive
Substance Use	Molina, 2012 [234]	African American adults (3570) Asian American adults (2095) Caribbean Black adults (1621) Latino adults (2554) Non-Hispanic white adults (4180)	National (NSAL; CPES)	Substance use disorder (DSM-IV): WMH-CIDI assessed	Not applicable	Positive
Substance Use	Mays, 2018 [235]	African American men (1222) U.S.-born Caribbean Black men (176) Caribbean Black men (461)	National (NSAL)	Substance use disorder (DSM-IV): WMH-CIDI assessed	Mixed	Mixed
Substance Use	Nguyen, 2018 [236]	US-born Black adults (626) Foreign-born Black adults (41) US and foreign-born Asian; Latino populations	Nationals (HINTS)	Smoking: Self-reported status	Positive	Positive
Substance Use	Saint-Fort, 2019 [237]	US-born Black adults (43,560) Africa-born Black adults (1911) West-Indies-born Black adults (2194) Europe-born Black adults (192)	National (US Census)	Smoking: Self-reported status	Mixed	Mixed
Substance Use	Jones, 2020 [238]	US-born Black women (2242) U.S. born Caribbean women (264) Foreign-born Caribbean Black women (705)	National (NSAL)	Substance use disorder (DSM-IV): WMH-CIDI assessed	Positive	Positive
Substance Use	Jegede, 2021 [239]	US-born Black adults w/one immigrant parent (441) U.S.-born Black adults (6683) Caribbean-born Black adults (327) African-born Black adults (218)	National (NESARC-III)	Substance use disorder (DSM-V): WMH-CIDI assessed	Positive	Positive
Substance Use	Okpala, 2022 [240]	US-born Black adults (n/a) Foreign-born Black adults (n/a) US and foreign-born Asian; Latino populations	National (NHIS)	Cigarette use: Self-reported	Positive	Positive
Substance Use	Cano, 2022 [241]	US born Black adults (56,233) Foreign born Black adults (2218) US and foreign-born White; Asian; Latino populations	National (NCHS)	Drug overdose mortality	Positive	Positive
HRQoL: Self-report	Lucas, 2003 [59]	African American adults (13,921) Foreign-Born Black adults (1486)	National (NHIS)	Self-rated health status: Self-reported item	Positive	Positive
HRQoL: Self-report	Read, 2005 [17]	African American adults (24,540) Foreign-born Black adults (2931)	National (NHIS)	Self-rated health: Self-reported physical health Activity limitation: Self-reported	Positive	Positive
HRQoL: Self-report	Antecol, 2006 [187]	African American adults (35,642) Foreign-born Black adults (2446) US and foreign-born White; Latino populations	National (NHIS)	Self-rated health: Self-reported physical health Activity limitation: Self-reported	Positive	Positive
HRQoL: Self-report	Singh, 2006 [22]	African American adults (n/r) Foreign-born Black adults (n/r) US and foreign-born White; Asian; Latino populations	National (US Census; NHIS)	Self-rated health: Self-reported	Positive	Not applicable
HRQoL: Self-report	Elo, 2008 [68]	African American adults (22,545) Hispanic-born Black adults (283) Caribbean/South American-born Black adults (1485) African-born Black adults (574) European-born Black adults (93)	National (US Census; NHIS)	Self-rated health: Self-reported chronic conditions	Positive	Positive
HRQoL: Self-report	Keane, 2009 [242]	African American adults (50) Black Caribbean adults (50)	Florida	Self-rated health: 8-item SF Health Survey	Positive	Positive
HRQoL: Self-report	Acevedo-Garcia, 2010 [243]	First-Gen Black adults (6244) Second-Gen Black adults (1306) Third-Gen Black adults (61,391)	National (US Census)	Self-rated: Reported physical health	Positive	Positive
HRQoL: Self-report	Griffith, 2011 [244]	African American adults (3570) US-born Caribbean Black adults (440) Caribbean-born Black adults (1166)	National (NSAL)	Self-rated: Reported physical health	Positive	Negative
HRQoL: Self-report	Hamilton, 2011 [14]	African-born adults (2128) South American-born Black adults (609) Caribbean-born Black adults (4333) European-born Black adults (164) Central American-born Black adults (945)	National (US Census)	Self-rated health: Self-reported measures	Positive	Positive
HRQoL: Self-report	Krieger, 2011 [57]	African American adults (193) Foreign-born Black adults (275)	Boston, MA	Self-rated health: 12-item SF Health Survey	Positive	Positive
HRQoL: Self-report	Erving, 2016 [245]	African American adults (3138) US-born Caribbean Black adults (417) Caribbean-born Black adults (1033)	National (NSAL)	Self-rated health: Self-reported, chronic, acute	Positive	Not applicable
HRQoL: Self-report	Hamilton, 2014 [44]	African American adults (4292) Foreign-born Black adults (264)	National (US Census)	Health advantage: Self-reported measure	Positive	Positive
HRQoL: Self-report	Christie-Mizell, 2017 [246]	African American adults (2960) US-born Caribbean Black adults (311) Caribbean-born Black adults (820)	National (NSAL)	Self-rated health: Self-reported	Positive	Mixed
HRQoL: Self-report	Maskileyson, 2021 [247]	US born Black adults (21,185) Foreign born Black adults (2967) US and foreign-born White; Asian; Latino populations	National (NHIS)	Self-rated: Reported physical health	Positive	Mixed
HRQoL: Self-report	Erving, 2022 [248]	African-American women (644) Afro-Caribbean women (223)	National (NSAL)	Self-rated: Reported physical health	Positive	Positive
HRQoL: General	Singh, 2002 [249]	African American adults (25,655) Foreign-born Black adults (777)	National (NLMS)	All-cause mortality: Combined risk of mortality from all major causes	Positive	Positive
HRQoL: General	Singh, 2004 [20]	African American adults (n/r) Foreign-born Black adults (n/r) US and foreign-born Asian and Hispanic groups	National (NVSS; NHIS; US Census)	Mortality risk: Risk of mortality from all major causes	Positive	Positive
HRQoL: General	Singh, 2006 [22]	African American adults (n/r) Foreign-born Black adults (n/r) US and foreign-born White; Asian; Latino populations	National (US Census; NHIS)	Life expectancy: Death records Childhood obesity: Prevalence rate	Positive	Positive
HRQoL: General	Singh, 2013 [21]	African American adults (n/r) Foreign-born Black adults (n/r)	National (NVSS; NHIS)	Life expectancy: Death records Childhood obesity: Prevalence rate	Positive	Not applicable
HRQoL: Aging	Singh, 2002 [249]	African American adults (3318) Foreign-born Black adults (40)	National (NLMS)	All-cause mortality: Combined risk of mortality from all major causes	Positive	Positive
HRQoL: Aging	Jackson, 2005 [16]	African American adults (3430) Foreign-born Black adults (1167)	National (NSAL)	Self-reported health: self-reported Mental disorders: DSM-IV assessed	Positive	Not applicable
HRQoL: Aging	Doamekpor, 2015 [250]	African American adults (2745) Foreign-born Black adults (152)	National (NHANES)	Allostatic load score: comprised systolic blood pressure, diastolic blood pressure, c-reactive protein, high-density lipoprotein, total cholesterol, creatinine clearance, serum albumin)	Positive	Positive

Positive: Black immigrants have a better health outcome than African Americans; Negative: Black immigrants have a worse health outcome than African Americans; Mixed: the disparities in health outcomes are not conclusive; Not applicable: the test was not conducted/reported. Abbreviations: AUDIT, Alcohol Use Disorders Identification Test; WMH-CIDI, World Mental Health—Compositive International Diagnostic Interview.

## Data Availability

Not applicable.
